# Taurine Protects against the Fatty Liver Hemorrhagic Syndrome in Laying Hens through the Regulation of Mitochondrial Homeostasis

**DOI:** 10.3390/ijms241210360

**Published:** 2023-06-20

**Authors:** Jishuang San, Jianmin Hu, Huiping Pang, Wenjun Zuo, Na Su, Zimeng Guo, Gaofeng Wu, Jiancheng Yang

**Affiliations:** Liaoning Provincial Key Laboratory of Zoonosis, College of Animal Science & Veterinary Medicine, Shenyang Agricultural University, Shenyang 110866, China; sanjishuang_syau@163.com (J.S.); hujianmin59@163.com (J.H.); panghuiping@sinochem.com (H.P.); zwjddeyx@163.com (W.Z.); 18842456311@163.com (N.S.); guozimeng297@163.com (Z.G.)

**Keywords:** taurine, laying hens, fatty liver hemorrhagic syndrome, mitochondrial, autophagy

## Abstract

Metabolic-associated fatty liver disease (MAFLD) is a chronic liver disease caused by fat deposition in the liver of humans and mammals, while fatty liver hemorrhagic syndrome (FLHS) is a fatty liver disease in laying hens which can increase the mortality and cause severe economic losses to the laying industry. Increasing evidence has shown a close relationship between the occurrence of fatty liver disease and the disruption of mitochondrial homeostasis. Studies have proven that taurine can regulate hepatic fat metabolism, reduce hepatic fatty deposition, inhibit oxidative stress, and alleviate mitochondrial dysfunction. However, the mechanisms by which taurine regulates mitochondrial homeostasis in hepatocytes need to be further studied. In this study, we determined the effects and mechanisms of taurine on high-energy low-protein diet-induced FLHS in laying hens and in cultured hepatocytes in free fatty acid (FFA)-induced steatosis. The liver function, lipid metabolism, antioxidant capacity, mitochondrial function, mitochondrial dynamics, autophagy, and biosynthesis were detected. The results showed impaired liver structure and function, mitochondrial damage and dysfunction, lipid accumulation, and imbalance between mitochondrial fusion and fission, mitochondrial autophagy, and biosynthesis in both FLHS hens and steatosis hepatocytes. Taurine administration can significantly inhibit the occurrence of FLHS, protect mitochondria in hepatocytes from disease induced by lipid accumulation and FFA, up-regulate the expression levels of Mfn1, Mfn2, Opa1, LC3I, LC3II, PINK1, PGC-1α, Nrf1, Nrf2, and Tfam, and down-regulate the expression levels of Fis1, Drp1, and p62. In conclusion, taurine can protect laying hens from FLHS through the regulation of mitochondrial homeostasis, including the regulation of mitochondrial dynamics, autophagy, and biosynthesis.

## 1. Introduction

Nonalcoholic fatty liver disease (NAFLD), which has been renamed as metabolic-associated fatty liver disease (MAFLD) in recent years [1], is a common chronic disease characterized by fatty deposits in hepatic cells and liver steatosis [2]. Liver steatosis is mainly characterized by an imbalance between fat secretion and metabolism after the transportation of fat to the liver [3]. In recent years, the combination of image-based digital liver biopsy with liver histology and liquid biopsy could yield a new multimodal approach to the study of the human fatty liver in clinical pathology [4]. In addition, fatty liver disease has become the disease with the highest incidence of chronic liver disease worldwide, the incidence of which in China is approximately 15–40% [5,6,7]. In Korea, the incidence of fatty liver disease (per 1000 person-years) is 42.8 overall [8]. It was also estimated by the United States that by 2030, the number of fatty liver disease cases will be approximately 100 million [9]. In addition to human and mammalians, fatty liver disease can also occur in high-yield laying hens, named fatty liver hemorrhagic syndrome (FLHS), the incidence of which can be as high as 30%, seriously causing enormous economic loss that affects the poultry industry [10]. FLHS is a metabolic disease characterized by a lipid metabolism disorder and accompanied by individual obesity, a decreased egg production rate, abdominal or subcutaneous fat accumulation, or even death induced by internal bleeding [11]. The pathogenesis of FLHS is the imbalance of lipid metabolism caused by various factors such as nutrition, metabolism, environment, hormone and heredity, and the pathogenesis of fatty liver disease is similar to that of FLHS [12]. Especially in birds, the liver is a crucial organ for lipid metabolism. Mitochondria play a crucial role in lipid catabolism and anabolism in addition to acting as a power plant of the cell. During these processes, reactive oxygen species (ROS) are produced within the mitochondria. The excessive accumulation of ROS increases the permeability of the mitochondrial outer membrane (MOMP), decreases mitochondrial membrane potential (MMP), and ultimately destroys mitochondrial integrity [13] and homeostasis, which are key cellular biological processes involved in controlling mitochondrial quality, function, shape, apoptosis signal transduction, and cell survival [14]. Mitochondrial fusion, fission, autophagy, and biosynthesis work together to maintain mitochondrial homeostasis. Numerous studies have shown that mitochondrial damage and dysfunction are closely related to fatty liver disease, which may be caused by abnormal mitochondrial dynamics defined as continual fusion and division [15,16]. Meanwhile, damaged mitochondria participate in mitochondrial autophagy (mitophagy), which plays a crucial role in NASH by regulating lipid droplet accumulation (lipophagy) [17,18]. The loss of mitochondria can be compensated with mitochondrial biosynthesis, and maintaining the number, function, and integrity of mitochondria has been shown to be a new promising treatment for liver disease [19]. Therefore, reducing hepatic mitochondrial damage and maintaining mitochondrial homeostasis are essential to ensuring normal fat metabolism in the liver and further preventing fatty liver disease.

Taurine, one of the few free amino acids, is named due to its first being identified and isolated from the bile of cattle. Taurine exerts many physiological and pharmacological effects, including anti-oxidation, cell membrane stabilization, detoxification, osmotic pressure regulation, brain and retina development [20]. Taurine has been reported to prevent hepatic fat metabolism disorders caused by various factors not only in mammals [21,22,23,24] but also in laying hens [25]. Our previous studies in rats with alcoholic liver disease also revealed the regulatory effects of taurine on hepatic lipid metabolism [26,27], the mechanism of which involves its anti-oxidative, anti-inflammatory, and anti-apoptotic effects [28,29]. Taurine can also protect mitochondrial damage from a variety of hazardous influences [30] by reducing mitochondrial swelling [31], maintaining the mitochondrial membrane potential [32], and increasing mitochondrial ATP production [31]. Taurine transporter knockout mice with taurine deficiency were observed to exhibit severe steatosis, which may be the consequence of the disruption to the mitochondrial structure and function [33]. Thus, we speculated that taurine might protect laying hens from FLHS through anti-oxidative and protective effects on mitochondria, especially maintaining mitochondrial homeostasis.

In this study, taurine was administered to both laying hens with FLHS, and steatosis hepatocytes were cultured in vitro to detect the protective effects of taurine on FLHS and to reveal its mechanism from the mitochondrial perspective.

## 2. Results

### 2.1. Effects of Taurine on Liver Index, Pathological Anatomy Changes and Incidence of FLHS

**Figure 1:**
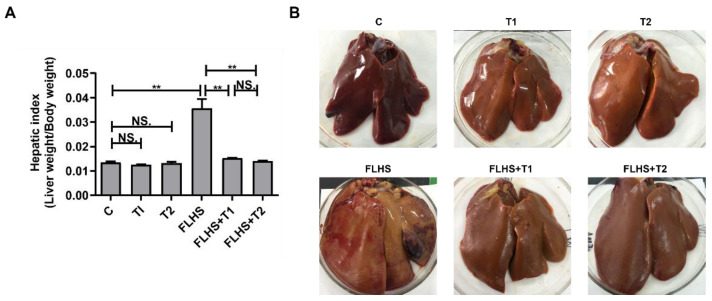
Effects of taurine on liver index and pathological anatomy changes. (**A**) Effects of taurine on liver index of laying hens. (**B**) Effects of taurine on pathological anatomy changes of laying hens. Data are described as the mean ± SEM. Not significant (NS.) *p* > 0.05, ** *p* < 0.01.

The liver index was determined by the ratio of the hepatic weight to the body weight of laying hens (Figure 1A). The liver index in the model group was significantly increased compared with that in the normal control group (*p* < 0.01). The liver index of hens with FLHS decreased significantly by taurine administration (*p* < 0.01). There were no differences between the normal control and taurine control groups, which were similar to the taurine prevention groups (*p* > 0.05). It can be seen from the figures of liver pathological anatomy that the liver of the normal control group was smooth and glossy, and the liver was compact, showing a normal dark red color and no bleeding. The liver of the model group was yellow, soft and fragile, and there were large blood clots or bleeding spots in the liver. In the taurine prevention groups, the liver returned to normal appearance with only a small amount of bleeding points exist locally (Figure 1B). The incidence of FLHS was calculated according to the situation of liver hemorrhage in each group, and the incidence of FLHS in the model group was as high as 70%, indicating that the FLHS model of laying hens was successfully established by using a high-energy and low-protein diet in this experiment. The incidence of FLHS in the taurine prevention groups was reduced to 10–12%, indicating that dietary taurine can effectively alleviate the occurrence of FLHS, which is basically consistent with the expected effect of the experiment (Table 1).

### 2.2. Screening the Concentrations of Taurine and FFA In Vitro

**Figure 2:**
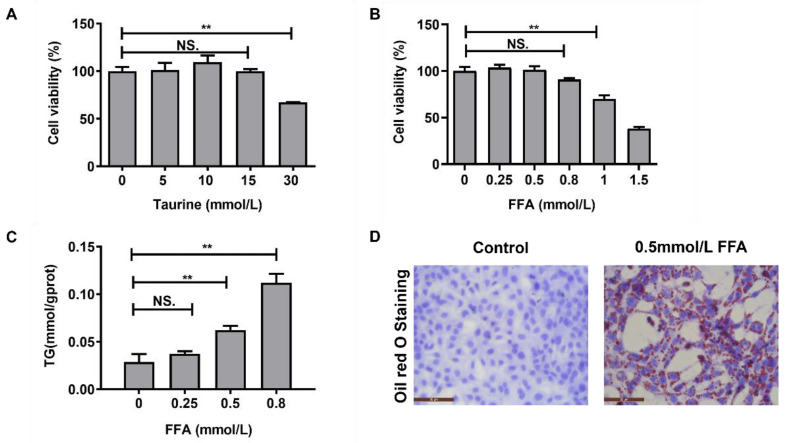
Results of the FFA and taurine concentration selection. (**A**) Cell viability under different concentrations of taurine. (**B**) Cell viability under different concentrations of FFA. (**C**) Effects of different concentrations of FFA on TG in the LO2 cells. (**D**) Oil red O staining of the LO2 cells in the normal group and the 0.5 mmol/L model group. NS. *p* > 0.05, ** *p* < 0.01.

LO2 cells were cultured with 0, 5, 10, 15, and 30 mmol/L taurine for 24 h. As shown in Figure 2A, the cell activity increased with increasing taurine concentration from 0 to 10 mmol/L but decreased with increasing taurine concentration from 10 to 30 mmol/L. Therefore, 10 mmol/L taurine was selected for subsequent tests. Similarly, different concentrations of FFA (0–1.5 mmol/L) were administered for 24 h to establish steatosis models. As shown in Figure 2B, there were no significant differences in cell viability at concentrations of 0, 0.25 to 0.5, and 0.8 mmol/L FFA. However, cell viability decreased significantly under the concentrations of 1 and 1.5 mmol/L FFA (*p* < 0.01). Therefore, 0–0.8 mmol/L FFA was administered to the LO2 cells, which was followed by the determination of TG. Figure 2C illustrates that the TG concentration increased significantly at 0.5 and 0.8 mmol/L FFA compared with 0 mmol/L FFA (*p* < 0.01). Oil red O staining was further carried out to verify the effects of 0.5 mmol/L FFA on LO2 cells. There were a large number of red lipid droplets in LO2 cells treated with 0.5 mmol/L FFA, as shown in Figure 2D, indicating that 0.5 mmol/L FFA was sufficient to induce obvious steatosis in the LO2 cells without affecting cell viability.

### 2.3. Effects of Taurine on Liver Function, Lipid Parameters, and Antioxidant Capacity Both In Vivo and In Vitro

**Figure 3:**
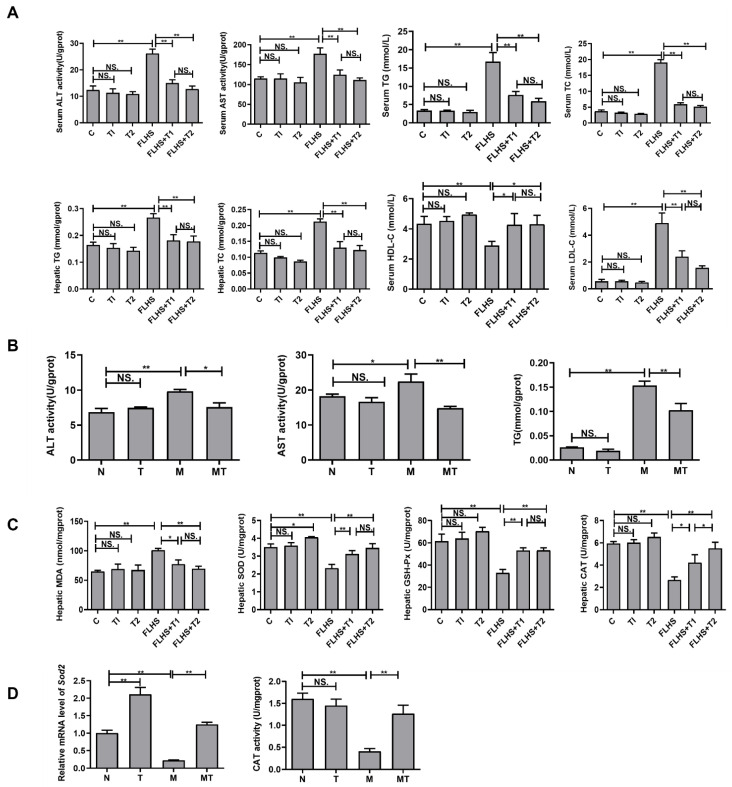
Effects of taurine on liver function, lipid parameters, and antioxidant capacity. (**A**) Effects of taurine on liver function and lipid parameters of laying hens. (**B**) Effects of taurine on liver function and lipid parameters in the LO2 cells. (**C**) Effects of taurine on antioxidant capacity in laying hens. (**D**) Effects of taurine on antioxidant capacity of the LO2 cells. Data are described as the means ± SEM (*n* = 6). NS. *p* > 0.05, * *p* < 0.05, ** *p* < 0.01.

As illustrated in Figure 3A, compared with laying hens in the normal control group, the serum hepatic function indices ALT and AST, serum lipid parameters TC, TG and LDL-C, and hepatic lipid parameters TC and TG were all significantly increased in the FLHS laying hens, while serum HDL-C was significantly decreased (*p* < 0.01). Compared with the FLHS groups, serum ALT, AST, TC, TG, and LDL-C, hepatic TC, and TG in taurine prevention groups were all significantly decreased (*p* < 0.01), while serum HDL-C levels were increased even further (*p* < 0.05). Meanwhile, the activities of ALT and AST and the concentrations of TG in the LO2 cells cultured with FFA were also significantly increased compared with the cells in the normal control group (*p* < 0.05 or *p* < 0.01), while taurine administration to the steatosis model cells significantly decreased ALT, AST, and TG (*p* < 0.05 or *p* < 0.01) (Figure 3B). None of the above results showed significant differences between the taurine control groups and the normal control group (*p* > 0.05). The above results indicated that taurine could protect hepatic cells and regulate lipid metabolism in both FLHS hens and steatosis LO2 cells cultured in vitro.

The antioxidant ability of hens was detected and is shown in Figure 3C. The hepatic MDA content was significantly increased, while the hepatic SOD, GSH-Px, and CAT activities were significantly decreased in the FLHS hens compared with the normal hens (*p* < 0.01). In the taurine prevention groups, the hepatic MDA content was significantly lower, while the hepatic SOD, GSH-Px, and CAT activities were significantly higher than those in the FLHS group (*p* < 0.05 or *p* < 0.01). Similar results were observed in vitro, as shown in Figure 3D. The expression levels of *Sod2* and the activity of CAT in the steatosis LO2 cells cultured with FFA were decreased, while taurine intervention significantly increased *Sod2* expression and CAT activity. These results suggested an augmentation effect of taurine on hepatic antioxidant capacity both in vivo and in vitro.

### 2.4. Effects of Taurine on Pathological Changes and Lipid Accumulation of Hepatocytes Both In Vivo and In Vitro

**Figure 4:**
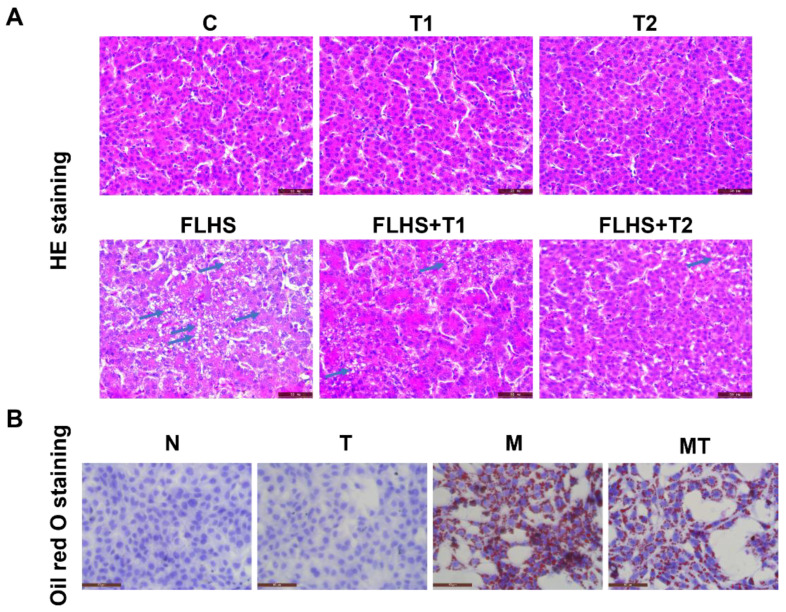
Effects of taurine on pathological changes and lipid accumulation in hepatocytes. (**A**) Representative images of H&E-stained liver tissues of the laying hens (magnification: 400×). The blue arrows indicate pathological changes. (**B**) Representative images of oil red O-stained LO2 cells (magnification: 400×).

H&E staining of the liver in laying hens was shown in Figure 4A. The liver lobules in the normal control and the taurine control hens had a normal structure, cell sizes were uniform, and cell cords were clear without lipid droplets. The FLHS hens exhibited more lipid droplets and inflammation in the liver than the normal control hens. Although there were still lobular structures in the FLHS hens, the arrangement of hepatocytes was disordered, and a large amount of granular degeneration and steatosis was observed. Meanwhile, karyolysis and necrosis were observed in the FLHS hens. In taurine preventive groups, liver injury and steatosis were much alleviated compared to the FLHS model hens. Oil red O staining in the LO2 cells cultured in vitro is shown in Figure 4B. Severe steatosis occurred in the model group, with a large number of red lipid droplets, while in the taurine intervention groups, there were fewer red lipid droplets than in the model group. The histological results showed that taurine could significantly ameliorate steatosis both in the FLHS hens and the hepatocytes cultured in vitro.

### 2.5. Effects of Taurine on Mitochondrial Function Both In Vivo and In Vitro

**Figure 5:**
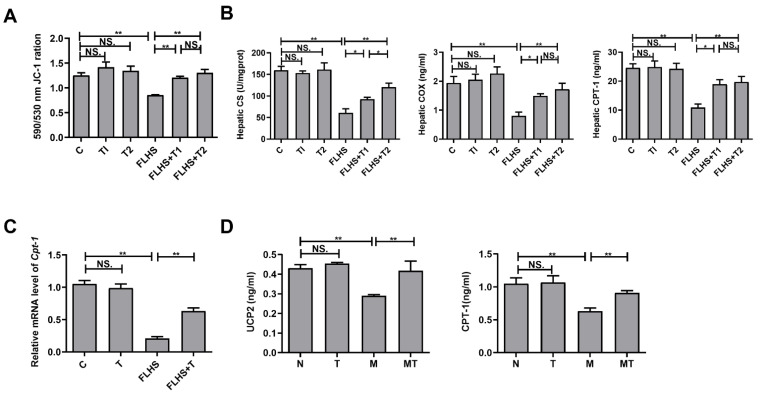
Effects of taurine on mitochondrial function. (**A**) Effects of taurine on the mitochondrial membrane potential in the liver of the laying hens. (**B**) Effects of taurine on mitochondrial marker enzymes in the liver of the laying hens. (**C**) Effects of taurine on the mRNA expression level of mitochondrial marker enzymes in the liver of the laying hens. (**D**) Effects of taurine on mitochondrial marker enzymes in the LO2 cells. Data are described as the means ± SEM. NS. *p* > 0.05, * *p* < 0.05, ** *p* < 0.01.

The MMP in the liver of the laying hens was detected by JC-1, as shown in Figure 5A. The MMP in the model group decreased significantly compared with that in the normal control and taurine prevention groups (*p* < 0.01), while there were no obvious differences between the normal control and the taurine control groups (*p* > 0.05), indicating that taurine can significantly increase the MMP of FLHS hens. Meanwhile, CS, COX, and CPT-1 activities were also assayed, and the results in Figure 5B showed that the activities of these enzymes decreased significantly in the FLHS hens compared with the normal control group (*p* < 0.01) and taurine prevention groups (*p* < 0.05 or *p* < 0.01). In the above experiments, the indexes of the taurine prevention groups were significantly different from the FLHS model group, while there was almost no significant difference between the two taurine prevention groups. At the same time, there was no significant difference among the taurine control groups and the normal control group. Therefore, in the following experiments, 0.05% taurine was administered in the taurine control and taurine prevention groups. Compared with the normal control group, the mRNA expression of *Cpt1* in the FLHS hens was significantly decreased (*p* < 0.01), while the mRNA expression of *Cpt1* was remarkably up-regulated by taurine administration in the taurine prevention group (*p* < 0.01), as shown in Figure 5C. The results indicated that taurine could increase the mitochondrial function of FLHS hens. Mitochondrial function was also detected in vitro, and Figure 5D shows that the activities of CPT-1 and the content of UCP2 in the LO2 cells cultured with FFA were significantly decreased compared with those in the normal control cells (*p* < 0.01). After taurine was administered to FFA-cultured LO2 cells, the expression of these parameters was significantly increased compared with the model group (*p* < 0.01), suggesting that taurine can also increase the mitochondrial function of hepatocytes cultured with FFA in vitro.

### 2.6. Effects of Taurine on Mitochondrial Homeostasis Both In Vivo and In Vitro

#### 2.6.1. Effects of Taurine on Mitochondrial Dynamic Balance

**Figure 6:**
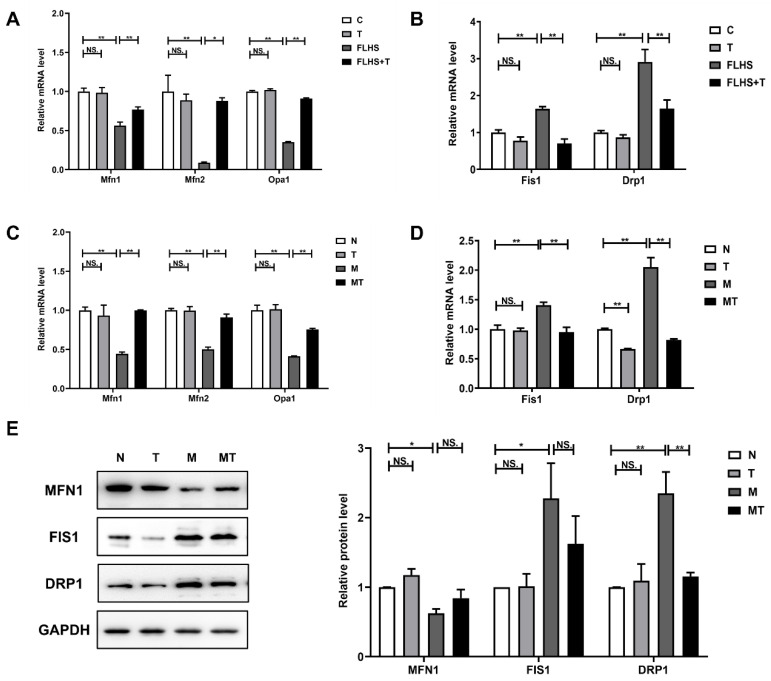
Effects of taurine on mitochondrial dynamic balance. (**A**) mRNA expression of mitochondrial fusion-related factors in the livers of the laying hens. (**B**) mRNA expression of mitochondrial fission-related factors in the livers of the laying hens. (**C**) mRNA expression of mitochondrial fusion-related factors in the LO2 cells. (**D**) mRNA expression of mitochondrial fission-related factors in the LO2 cells. (**E**) The expression of mitochondrial dynamic-related factors, MFN1, FIS1 and DRP1 in the LO2 cells were determined by Western blot analysis. Data are described as the means ± SEM. NS. *p* > 0.05, * *p* < 0.05, ** *p* < 0.01.

The effects of taurine on mitochondrial dynamics in the FLHS hens were manifested by the mRNA expression of *Mfn1*, *Mfn2,* and *Opa1*, which are mitochondrial fusion proteins, and the mRNA expression of *Fis1* and *Drp1*, which are mitochondrial fission proteins. The results are shown in Figure 6A,B. The mRNA expression of *Mfn1*, *Mfn2,* and *Opa1* in FLHS hens was significantly decreased compared with that in the normal control hens (*p* < 0.01) and the taurine preventive hens (*p* < 0.05 or *p* < 0.01), while the mRNA expression of *Fis1* and *Drp1* in the FLHS hens was significantly higher than that in the normal control and the taurine preventive groups (*p* < 0.01). The results illustrated that taurine could maintain the balance of mitochondrial dynamics by regulating hepatic mitochondrial fusion and fission in the livers of FLHS hens. This effect of taurine was also verified in vitro, as illustrated in Figure 6C,D. *Mfn1*, *Mfn2,* and *Opa1* mRNA expression in steatosis LO2 cells was also significantly lower, while the mRNA expression levels of *Fis1* and *Drp1* were much higher than those in the control (*p* < 0.01) and the taurine groups (*p* < 0.01). Meanwhile, the protein levels of MFN1, FIS1, and DRP1 were also examined, and the change trends in these protein levels shown in Figure 6E were the same as those in mRNA expression, further indicating that taurine administration can also regulate the mitochondrial dynamic balance of steatosis hepatic cells cultured in vitro.

#### 2.6.2. Effects of Taurine on Mitochondrial Autophagy

**Figure 7:**
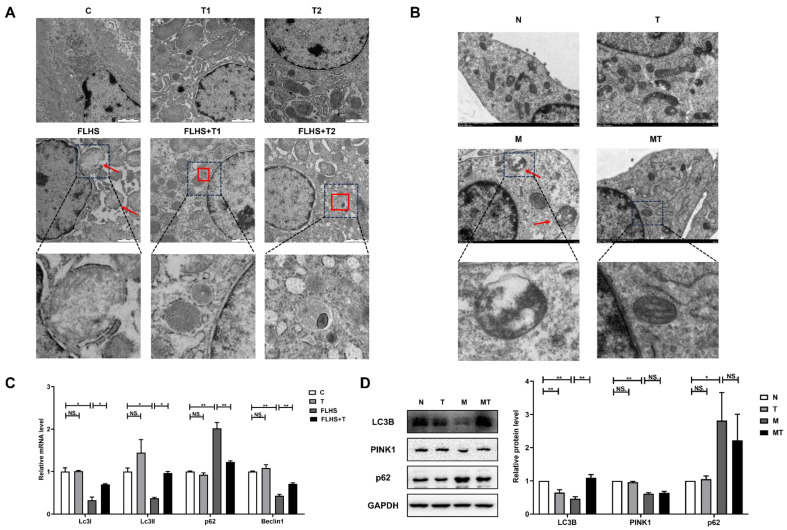
Effects of taurine on mitochondrial autophagy. (**A**) Ultrastructure examination of mitochondria and autophagosomes in the livers of the FLHS hens under transmission electron microscopy (magnification: 1 μm). The red arrows indicate disrupted mitochondria; the red boxes represent autophagosomes. (**B**) Ultrastructure examination of mitochondria and autophagosomes in the LO2 cells under transmission electron microscopy (magnification: 2 μm). The red arrows indicate disrupted mitochondria. (**C**) mRNA expression of mitochondrial autophagy-related factors in the livers of the FLHS hens. (**D**) The expression of mitochondrial autophagy-related factors, LC3B, Pink1 and p62 in the LO2 cells were determined by Western blot analysis. Data are described as the means ± SEM. NS. *p* > 0.05, * *p* < 0.05, ** *p* < 0.01.

Mitochondrial autophagy in the laying hens and the LO2 cells was observed by electron microscopy in vivo and in vitro. The results showed that normal mitochondria were observed in the normal control hens and the LO2 cells with clear mitochondrial contours and mitochondrial crests, as shown in Figure 7A,B. Autophagosomes could be found inside the cells. In the liver of the FLHS hens and the steatosis LO2 cells, the mitochondria were abnormal in morphology, as manifested by mitochondrial swelling, fuzzy contours, and unclear mitochondrial crests. In the taurine prevention groups both in vivo and in vitro, the mitochondrial injury was improved compared with that in the model groups. The mRNA expression levels of *Lc3I*, *Lc3II,* and *Beclin1* in the livers of the FLHS hens were significantly lower than those in the livers of the normal control hens (*p* < 0.05 or *p* < 0.01) and the taurine prevention hens (*p* < 0.05 or *p* < 0.01), while *p62* was significantly increased in the FLHS hens compared with the other groups (*p* < 0.01) (Figure 7C). The effect of taurine on mitochondrial autophagy was also detected by the protein levels of LC3B, PINK1, and p62 in the steatosis LO2 cells. Figure 7D shows that the protein expression levels of LC3B and PINK1 in the steatosis LO2 cells were significantly lower than those in the normal control group (*p* < 0.01), while taurine administration to the steatosis LO2 cells up-regulated the protein expression levels of LC3B and PINK1, and it down-regulated the protein expression of p62, indicating that taurine administered to both the FLHS hens and the steatosis LO2 cells can regulate mitochondrial autophagy.

#### 2.6.3. Effects of Taurine on Mitochondrial Biosynthesis

**Figure 8:**
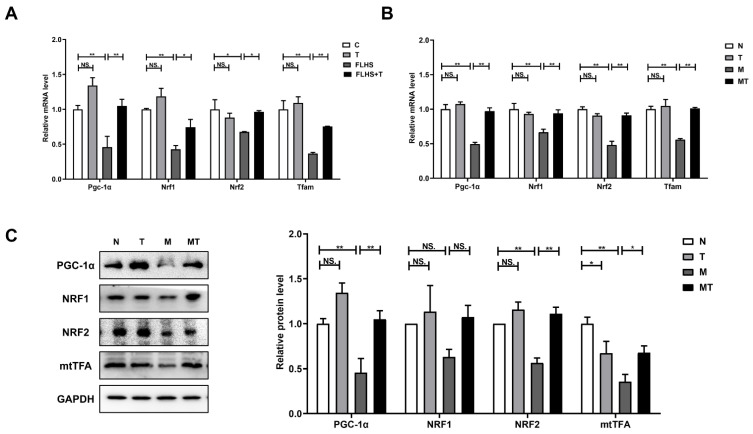
Effects of taurine on mitochondrial biosynthesis. (**A**) mRNA expression of mitochondrial biosynthesis-related factors in the livers of the FLHS hens. (**B**) mRNA expression of mitochondrial biosynthesis-related factors in the LO2 cells. (**C**) The expression of mitochondrial biosynthesis-related factors, PGC-1α, NRF1, NRF2, and mtTFA in the LO2 cells were determined by Western blot analysis. Data are described as the means ± SEM. NS. *p* > 0.05, * *p* < 0.05, ** *p* < 0.01.

PGC-1α, NRF1, NRF2, and TFAM, which have been reported as key promoters of mitochondrial biogenesis, were detected both in vivo and in vitro. Figure 8A showed that compared with the normal control hens, mRNA expression levels of *Pgc1α*, *Nrf1*, *Nrf2,* and *Tfam* in the FLHS hens were significantly decreased (*p* < 0.05 or *p* < 0.01) and were significantly up-regulated by taurine administration to FLHS hens (*p* < 0.05 or *p* < 0.01). The results in Figure 8B,C showed the expression of key factors for mitochondrial biosynthesis in the cultured LO2 cells. Both the mRNA and protein expression levels of PGC-1α, NRF1, NRF2, and mtTFA in steatosis cells were down-regulated compared with those in the control cells and the steatosis LO2 cells administered taurine. The results indicated that hepatic mitochondrial biosynthesis was inhibited in the FLHS hens and the steatosis LO2 cells and that taurine can promote mitochondrial biosynthesis both in vivo and in vitro.

## 3. Discussion

Nonalcoholic fatty liver disease is divided into early and late stages. The early stage is manifested by simple fatty liver disease, which is often ignored and neglected but can lead to further cirrhosis and hepatocellular carcinoma (HCC) in severe cases. Therefore, it has become increasingly urgent to study the pathogenesis and targets of early MAFLD. Similarly, FLHS is a common nutritional metabolic disease in laying hens during the peak laying period caused by the imbalance of nutrient metabolism, which leads to a disorder of the fat metabolism in laying hens. A variety of factors have been reported to induce FLHS, and there are currently multiple methods to establish the FLHS model, including excessive feed intake through forced feeding [34,35] and the injection of estradiol into laying hens [36]. Recently, a large number of studies demonstrated that a high-energy low-protein diet, which is rich in carbohydrates and low in protein, can successfully induce FLHS because it can readily convert acetyl-CoA into excessive fat and that fat cannot be exported from the liver [37,38]. In the current study, corn and soybean meal with a metabolic energy of 12.85 MJ/kg and a crude protein of 13.51% was fed to laying hens for 16 weeks, successfully inducing FLHS, which was manifested by the histological changes, the liver function indices, and the lipid metabolism parameters of the liver. Under these feeding circumstances, lipid synthesis increased in the liver, while the insufficient protein content led to a lower formation of apolipoprotein that binds and transports fat from the liver and finally induced hepatic fat accumulation. In addition, the low protein resulted in reduced egg production and lowered fat consumption, which further increased the fat accumulation in the liver. Meanwhile, high-energy and low-protein diets can also lead to the abnormal oxidation of fatty acids in the liver, further aggravating liver steatosis.

In an in vitro experiment, a steatosis cell model was established by the administration of free fatty acids (FFAs) to LO2 cells. Palmitic acid (PA), which is a kind of saturated fatty acid, and oleic acid (OA), which is a kind of monounsaturated fatty acid, were the most abundant FFAs in the diet and the serum [39]. A high level of serum FFA is closely related to metabolic syndrome and fatty liver disease [40]. Previous studies have shown that fatty acids (especially higher levels of saturated fatty acids) can induce steatosis and apoptosis. Compared with single unsaturated fatty acids (e.g., OA), the primary hepatocytes of mice and HepG2 cells exposed to saturated FFAs (e.g., PA) showed more advanced steatosis and apoptosis [41]. According to previous studies, FFA with a ratio of OA to PA of 2:1 was administered in this present study and successfully established a simple steatosis cell model with obvious lipid droplets and increased contents of TG inside the cells [42,43,44]. The results were similar to the report that cultured LO2 cells in a medium with FFA of 0.5 mmol/L (OA:PA = 2:1), which did not cause cytotoxicity and apoptosis but induced obvious steatosis in cells [45]. Both animal and cell models were successfully constructed in the present study and provided a theoretical basis for further study of the pathogenesis of MAFLD.

The liver is the main organ capable of fat storage and metabolism. ALT and AST, which are enzymes synthesized in hepatocytes, are sensitive indices for liver function because an elevated serum level of the two enzymes is detected when they are released into the blood under the circumstances of liver injury. In the present study, serum concentrations of ALT and AST were significantly higher in the FLHS hens, suggesting the destruction of the liver cells and an increase in the membrane permeability that resulted in a large amount of transaminase spilling into the blood from the hepatocytes, which subsequently leads to changes in organelles and other structures. Similar results were also observed in the cultured hepatic cells, in which the concentrations of ALT and AST increased in the steatosis LO2 cells. Taurine inhibited the increase in ALT and AST caused by hepatocyte injury in both the FLHS hens and the steatosis LO2 cells. The results were consistent with numerous studies confirming a protective effect of taurine on the liver, including our previous studies in rats with alcoholic liver disease and cultured hepatic cells [26,27,46]. TG and TC are components of lipids, and the serum concentrations of these can be used as indicators of lipid metabolism. HDL-C and LDL-C are responsible for the transportation of cholesterol. HDL-C promotes the clearance of cholesterol from tissue cells into the liver to be further metabolized, while LDL-C carries cholesterol into tissue cells. In the present study, the concentrations of serum/hepatic TC and TG and serum LDL-C were significantly increased, while serum HDL-C was significantly decreased in the FLHS hens, indicating a disorder in lipid metabolism of the liver. It can be observed that taurine significantly reduced the concentrations of TC, TG and LDL-C while increasing the concentration of HDL-C in the FLHS hens, indicating that taurine restored the hepatic lipid metabolism in the FLHS hens. Meanwhile, in cultured steatosis LO2 cells, the increase in TG concentration was significantly inhibited by taurine administration. The results of the current study were consistent with previous studies showing that taurine was found to reduce plasma and hepatic concentrations of TC, TG, and LDL-C while increasing HDL-C levels in rats fed a high-cholesterol diet [47,48]. It has also been reported that taurine can reduce TG levels in an NAFLD model in vitro [49]. Furthermore, the onset of a lipid metabolism disorder and increased fatty acid accumulation lead to surges in free radicals and lipid peroxidation, during which a large number of free radicals exceed the upper limit of the hepatic antioxidant capacity and aggravate the oxidative stress of the hepatocytes, which is known as the “two-hit” theory in the pathogenesis of NAFLD. MDA is a lipid peroxidation product produced in the peroxidation process which can reflect the level of lipid peroxidation. SOD and GSH-Px are active enzymes that can scavenge free radicals, and CAT is an enzyme scavenger that removes H_2_O_2_ in the body and is one of the key enzymes in the biological defense system. In this study, the results in the FLHS hens illustrated that the hepatic contents of MDA increased significantly, while the hepatic activities of SOD, GSH-Px, and CAT decreased. Taurine administered to the FLHS hens reduced hepatic MDA and increased hepatic SOD, GSH-Px, and CAT. Meanwhile, the content of CAT and the mRNA expression level of *Sod2* in the steatosis LO2 cells decreased compared with the normal control and the taurine prevention groups, indicating an antioxidant capacity of taurine on both the FLHS hens and the steatosis LO2 cells. A similar result of decreased antioxidant capacity during fatty liver development was demonstrated in both the FLHS chickens and the NAFLD rats [50,51]. The antioxidant effect of taurine is consistent with a study on the liver and kidney of mice fed ethanol and on the liver of rats with NAFLD [24,52,53]. The above results confirmed that liver function, lipid metabolism, and antioxidant capacity were affected when MAFLD occurred and that taurine played a positive role in protecting liver function, enhancing antioxidant enzymes and inhibiting lipid peroxidation.

Mitochondria are highly sensitive to oxidative stress [54]. ROS and lipid peroxides generated during fatty liver development attack mitochondria. Since mitochondria are responsible for the β-oxidation of fatty acids in the liver, dysfunction and damage in mitochondria is an essential cause of MAFLD. Studies have found that fatty liver induced by different factors is accompanied by mitochondrial damage in hepatocytes, which is also the basis for the occurrence of various liver diseases [55]. It has been concluded that fatty liver is also a mitochondrial disease [56,57]. The MMP can reflect the integrity of the mitochondrial membrane and indirectly reflect the function of mitochondria, the decline of which is an early indicator of mitochondrial injury. CPT-1, which is located in the outer membrane of mitochondria, can catalyze the transportation of long-chain fatty acids into mitochondria to further participate in β-oxidation and is considered to be a rate-limiting enzyme in the process. COX, which is located in the mitochondrial inner membrane, is the key enzyme for electron transfer in the respiratory chain. Abnormal COX activity affects mitochondrial membrane permeability and the mitochondrial respiratory chain, resulting in mitochondrial energy metabolism dysfunction. CS, located in the mitochondrial matrix, affects the synthesis of fatty acids and participates in the redox process of fatty acids and is one of the key enzymes in cell metabolism. As a transmembrane protein of the mitochondrial inner membrane, UCP2 can regulate the mitochondrial proton pump and alleviate mitochondrial damage. It has been reported that MMP decreases during NASH [58] and that initial fat synthesis can be inhibited by regulating CPT-1 [59], thereby inhibiting the development of fatty liver disease. Other studies have shown that the activation of CPT-1 can reduce the serum levels of AST and ALT in steatosis patients [60]. In addition, in the early stage of steatosis, the up-regulation of UCP2 can inhibit a large amount of ROS damage and play a protective role in cells [61]. The results of the present study also showed that MMP and mitochondrial marker enzymes decreased when FLHS occurred in the hens. After the taurine intervention, MMP increased, which was accompanied by increases in hepatic mitochondrial functional markers, including CPT-1, COX, and CS. Meanwhile, in the cultured steatosis LO2 cells, a decline was observed in the activities of CPT-1 and UCP2, the activities of which were significantly enhanced after taurine administration. These results were also demonstrated by the mitochondrial morphological changes in the model group observed by electron microscopy during the occurrence of fatty liver. When fatty liver occurred, the mitochondrial morphology of the model group showed pathological changes, and there were no autophagosomes. Studies on the hepatocytes of mice with fatty hepatitis have shown that the endoplasmic reticulum ruptured, forming isolated membranes (phagosomes) that surround mitochondria, and that defective autophagy contributed to the development and progression of steatohepatitis [62]. The changes in the mitochondrial morphology were significantly improved after the taurine intervention. The above results showed that taurine could inhibit damage to the structure and function of hepatic mitochondria in the FLHS hens and the steatosis LO2 cells through the protection of the mitochondrial membrane integrity, permeability, the regulation of the β-oxidation of fatty acids, and the mitochondrial respiratory chain.

Studies have considered that mitochondrial homeostasis, including mitochondrial dynamics, autophagy, and biosynthesis, may be a further mechanism for regulating NASH [63,64] and that these consequently highlight potential therapeutic targets for this disease. The effects of the high-fat diet/FFA and the hypothetical role of taurine in the regulation of mitochondrial homeostasis were detected and discussed in the present study. Constant fusion and fission are called mitochondrial dynamics. Mitochondrial fusion includes mitochondrial inner membrane fusion and outer membrane fusion, which are regulated by different molecules. Mfn1 and Mfn2 are located on the mitochondrial outer membrane. The N-terminus of Mfn1 and Mfn2 is a conserved GTPase active domain, while the C-terminus is a coiled-coil domain, and the interactions of these coiled-coil domains promote mitochondrial outer membrane fusion [65]. OPA1 is located on the mitochondrial inner membrane and binds to phospholipids, causing GTP hydrolysis, promoting the recombination of lipid molecules on the mitochondrial membrane, and leading to mitochondrial inner membrane fusion [66]. At the same time, Drp1 is recruited to the mitochondrial outer membrane by Fis1 and is enriched in the potential cleavage sites, forming a ring structure around the mitochondria and changing the distance between molecules through GTP decomposition, and it gradually compresses until the mitochondria is fragmented into two independent mitochondria. This process is named mitochondrial fission. Then, Drp1 returns to the cytoplasm to regulate a new round of mitochondrial division [67]. In the present study, the decreased expression of Mfn1, Mfn2, and OPA1 was accompanied by a significant increased expression of FIS1 and Drp1 observed in the FLHS hens and the steatosis hepatic cells cultured in vitro, suggesting that the inhibition of mitochondrial fusion and the promotion of mitochondrial fission were induced by the fatty liver disease. These results were not completely surprising, since there were previous reports that the mRNA expression levels of *Mfn1*, *Mfn2,* and *OPA1* were significantly decreased in liver injury induced by a high-fat diet [68], while the expression of mitochondrial fission mediators, including DRP1 and FIS1, induced by HFD was further exacerbated in the livers of mice [69]. The expression of FIS1 and DRP1 was also up-regulated in steatosis HepG2 cells [70]. Taurine was found to up-regulate the expression of the mitochondrial fusion proteins Mfn1, Mfn2, and OPA1 while down-regulating the expression of the mitochondrial fission proteins FIS1 and Drp1 in both the FLHS hens and the steatosis LO2 cells, indicating a regulatory effect of taurine on the mitochondrial dynamic balance, which is essential for the maintenance of normal mitochondrial morphology and function.

Severely damaged mitochondria can be fragmented through abnormal fusion and division and cleared through mitochondrial autophagy. First, the autophagy precursor, which has a double-layer membrane, is formed in the cells and then gradually extends to wrap up the mitochondria to form autophagosomes, which is followed by fusion with the lysosome membrane to form autophagolysosomes and complete the mitophagy process. In this process, the isolation membrane extends under the action of Beclin1, a key autophagy protein, and gradually wraps the damaged organelles to form autophagosomes. The LC3 precursor (ProLC3) cleaves 22nd amino acids on the C-terminal 5-peptide catalyzed by ATG4B to form LC3-I and then covalently binds with phosphatidylethanolamine (PE) to form LC3-II catalyzed by ATG7. LC3-II is the structural protein of autophagosomes. When autophagosomes are combined with lysosomes, LC3-II located on the membrane of autophagosomes can be degraded by breaking the conjugated double bond with PE, so the LC3-II/LC3-I ratio can reflect the level of autophagy. In the meantime, LC3 also plays a role in mitophagy, regulating the number and quality of mitochondria by reducing mitochondria to basic levels to satisfy the cellular energy needs and prevent excessive ROS production. Mitochondrial autophagy is mediated by the PINK1/Parkin pathway through the initiation of PINK1, which degrades continuously under healthy circumstances. PINK1 remains stable when the mitochondria are damaged and recruits Parkin, which is an E3 ligase, to depolarize the mitochondria. This process induces mitochondrial ubiquitination, in the matrix of which P62 accumulates and binds to LC3 to initiate mitochondrial autophagy [71]. The present results showed a decrease in the expression of Beclin-1, PINK1, Parkin, LC3-I, and LC3-II in both the FLHS hens and steatosis hepatic cells, indicating that the autophagy scavenging mechanism was weakened by steatosis in the hepatic cells. These results were accompanied by previous studies in NAFLD mice showing that the expression of positive autophagy and mitophagy-related factors were all decreased, while p62 was increased [72]. Additionally, a decrease in autophagy levels was observed in male SD rats with fatty liver disease [73]. The up-regulation of autophagy was reported to be conducive to promoting the removal of hepatic lipids, while the inhibition of autophagy levels promoted lipid accumulation [74,75]. In this study, taurine was found to up-regulate the levels of autophagy and mitochondrial autophagy-related factors through the regulation of the PINK1/Parkin pathway. Therefore, the increased expression of mitophagy-positive regulatory proteins, after the taurine intervention, can be considered a compensatory mechanism for mitochondrial over-injury in the FLHS hens and the steatosis LO2 cells.

Meanwhile, new mitochondria were formed through mitochondrial biosynthesis that was conducted through the duplication, transcription, and translation of the mitochondrial genome to compensate for the damaged mitochondria and further maintain normal energy metabolism and mitochondrial homeostasis. Mitochondrial biosynthesis is mainly regulated by PGC-1α, which can activate the expression of TFAM by directly combining with NRF1 and NRF2 and promoting the translocation of TFAM to the mitochondria and multipoint binding with mtDNA, thereby regulating mtDNA transcription and replication [76]. In the present study, the levels of PGC-1α, NRF1, NRF2, and Tfam in the FLHS hens and the steatosis model cells decreased significantly, the contents of which were significantly up-regulated after the taurine intervention, which seemed to represent an adaptive response of taurine to stimulate mitochondrial and tissue/cell renewal. Since previous studies have concluded that increasing mitochondrial biosynthesis could be a new method to treat fatty liver disease [19], the present results suggested a positive effect of taurine on the PGC-1α-mediated mitochondrial biosynthesis mechanism and restored the number and function of mitochondria.

## 4. Materials and Methods

### 4.1. Hens, Diets, and Experimental Design

A total of 48 healthy 23-week-old Hy-Line brown laying hens with similar body weights and egg yields were obtained (average weight 1.5 kg ± 0.2 kg) and kept in three-layer cages. During the experiment, the hens were provided with feed twice a day (at 8:00 and 16:30) and water ad libitum. The laying hens were housed under a 16–8 h light–dark cycle at controlled temperature (18–21 °C) and humidity (40%–70%). Hens were randomly divided into six groups after one week of acclimation: hens in the normal control group (C) were fed a standard corn–soybean meal-based diet. Hens in the taurine control groups (T1 and T2) were provided a corn–soybean meal-based diet supplemented with 0.05% and 0.3% taurine, respectively. A high-fat and low-protein diet for hens fed the FLHS model group (FLHS). Hens in the taurine prevention groups (FLHS + T1 and FLHS + T2) were fed the same diet as hens in the FLHS group supplemented with 0.05% and 0.3% taurine, respectively. Hens were fed either a standard or high-fat and low-protein diet (Table 2). After 16 weeks of feeding, all hens were sacrificed, and blood and livers were collected for further analysis. This study was reviewed and approved by the Animal Care and Use Committee of Shenyang Agricultural University (2020090102).

### 4.2. Cell Culture and Treatments

LO2 cells were purchased from Shanghai Boquan Biotechnology Co., Ltd. (Shanghai, China), and the cells were cultivated in Roswell Park Memorial Institute (RPMI)-1640 medium (Gibco, Grand Island, NY, USA) containing 10% (*v*/*v*) fetal bovine serum (VivaCell, Shanghai, China), 100 U/mL penicillin, and 100 μg/mL streptomycin at 5% CO_2_, 37 °C. The cells were passaged for subculturing or subsequent experiments when they were 70–80% confluent. The cells were treated with free fatty acid (FFA) (oleic acid (OA): palmitic acid (PA) = 2:1) in serum-free media for 24 h to establish a steatosis cell model. Meanwhile, taurine was administered to the medium and cultured at 37 °C for 24 h. The cells were divided into four groups: the normal control group (N), the taurine control group (T), the model group (M), and the taurine intervention group (MT).

### 4.3. Cell Viability Assay

LO2 cells were seeded in 96-well plates and treated differently when the cells grew to 90% confluency. Then, Cell Counting Kit-8 (CCK-8; Solarbio, Beijing, China) was used to measure cell viability. First, 10 μL CCK-8 assay reagent was added to each well, which was followed by incubation at 37 °C for 4 h. Finally, the absorbance of the formazan solution was assessed using a microplate reader (Infinite M200 PRO, TECAN, Shanghai, China) at 450 nm.

### 4.4. Biochemical Analysis

All biochemical analyses were performed using commercial kits (Nanjing Jiancheng Bioengineering Institute, Nanjing, China). Lipid metabolism indices, including total triglycerides (TGs) (A110-1-1), total cholesterol (TC) (A111-1-1), high-density lipoprotein cholesterol (HDL-C) (A112-1-1) and low-density lipoprotein cholesterol (LDL-C) (A113-1-1); liver function indices, including alanine aminotransferase (ALT) (C009-2-1) and aspartate aminotransferase (AST) (C010-2-1); liver antioxidant capacity indices, including total superoxide dismutase (T-SOD) (A001-1-2), malondialdehyde (MDA) (A003-1-2) and glutathione peroxidase (GSH-Px) (A005-1-2); and mitochondrial function indices, including hydrogenase (CAT) (A007-1-1), citric acid synthase (CS) (A108-1-1), carnitine palmitoyltransferase-I (CPT-1) (H230), cytochrome C oxidase (COX) (H210) and coupling protein 2 (UCP2) (H548-1), were all measured according to the protocol of the manufacturer using an automatic biochemistry analyzer (Infinite M200 PRO, TECAN, Shanghai, China).

### 4.5. Hematoxylin and Eosin (H&E) Staining

Hepatic tissues from the exact location of the same lobe were fixed in 4% paraformaldehyde solution and embedded in paraffin. Five-micrometer-thick sections were cut and stained with H&E. The sections were then examined and photographed under 400× magnification using a Leica inverted microscope (DM4000B).

### 4.6. Oil Red O Staining

LO2 cells cultured in six-well plates were pretreated with FFA and taurine for 24 h. The cells washed with PBS were fixed with 4% formaldehyde for 10 min. Subsequently, the cells were stained with oil red O working solution for 15 min. The nuclei were stained with hematoxylin for another 1 min, and cell steatosis was observed under a light microscope (OLYMPUS, Tokyo, Japan) at 400× magnification.

### 4.7. Transmission Electron Microscopy (TEM)

The samples were fixed in 2.5% glutaraldehyde for TEM and dehydrated in ethyl alcohol and acetone. Then, the samples were embedded in epoxy resin. Ultrathin sections (thickness, 50–70 nm) were cut with a diamond knife and then double-stained with uranyl acetate and lead citrate. The ultra-microstructure of the samples was examined by a transmission electron microscope (HITACHI, Tokyo, Japan).

### 4.8. Real-Time Quantitative PCR (RT-qPCR)

The total RNA from the liver and LO2 cells was obtained by using a total RNA extraction kit (TIANGEN, Beijing, China, DP430). Then, cDNA was synthesized using the PrimeScript RT Reagent Kit with a gDNA Eraser according to the manufacturer’s instructions (Takara Bio, Dalian, China, RR047A). The expression levels of related genes were quantified by RT-qPCR using TB Green Premix Ex Taq II (Takara Bio, Dalian, China, RR820A). The sequences of all primers used for the RT–PCR are listed in Table 3. The relative gene expression levels were calculated using the 2^−ΔΔCt^ method [77]. The results are presented as relative fold changes in the value of the control group after normalizing to the endogenous control *β*-*actin*.

### 4.9. Western Blot

LO2 cells were lysed using the RIPA lysate (Beyotime, Shanghai, China). The protein concentration was determined using the bicinchoninic acid (BCA) method. Equal amounts (20 μg) of total proteins were separated by 10% sodium dodecyl sulfate–polyacrylamide gel electrophoresis (SDS–PAGE), which was followed by electrotransfer to polyvinylidene difluoride (PVDF) membranes. The membranes were blocked in 5% nonfat dried milk for 1 h at room temperature and then incubated with GAPDH (dilution 1:5000, Immunoway, Texas, USA, YM3029), mitofusin 1 (Mfn1) (dilution 1:1000, Abcam, Cambridge, UK, ab129154), fission protein 1 (Fis1) (dilution 1:1000, Abcam, Cambridge, UK, ab156865), dynamin-related protein 1 (Drp1) (dilution 1:1000, Abcam, Cambridge, UK, ab184247), microtubule-associated protein light chain 3B (LC3B) (dilution 1:1000, Abcam, Cambridge, UK, ab192890), PTEN-induced kinase 1 (PINK1) (dilution 1:1000, CST, Massachusetts, USA, 6946), p62 (dilution 1:1000, Abcam, Cambridge, UK, ab109012), peroxisome proliferator-activated receptor γ coactivator 1-α (PGC-1α) (dilution 1:1000, Abcam, Cambridge, UK, ab106814), nuclear respiratory factor 1 (NRF1) (dilution 1:1000, Abcam, Cambridge, UK, ab175932), nuclear respiratory factor 2 (NRF2) (dilution 1:1000, Abcam, Cambridge, UK, ab137550), mitochondrial transcription factor A (mtTFA) (dilution 1:1000, Abcam, Cambridge, UK, ab176558), and second antibodies (dilution 1:5000, Proteintech, Rosemont, PA, USA) overnight at 4 °C. The levels of GAPDH were used as loading controls. The bands were visualized with an enhanced chemiluminescent direct labeling (ECL) system. The optical density of each band was scanned and semi-quantified by using the Quantity One software (Bio–Rad, Hercules, CA, USA).

### 4.10. Statistical Analyses

All statistical analyses were performed using IBM SPSS Statistics. One-way analysis of variance (ANOVA) followed by a LSD post-hoc test was used to compare differences between groups. Data are expressed as the mean ± standard error of the mean (SEM). Differences were considered significant at *p* < 0.05.

## 5. Conclusions

In conclusion, the occurrence of fatty liver disease is related to mitochondrial injury and an imbalance in mitochondrial homeostasis. Taurine exerts a protective effect on hepatic cells and inhibits FLHS through the maintenance of mitochondrial homeostasis, including the balance of mitochondrial kinetics, autophagy, and biosynthesis, thereby reducing mitochondrial damage and further promoting the key transformation of liver tissue and cells. The results provide a possible target and a scientific basis for the application of taurine to prevent the occurrence of fatty liver disease and mitochondria-related diseases. Both the pathophysiological roles and actions of taurine in hepatocytes may be more complex than currently thought, and these roles need further clarification.

## Figures and Tables

**Table 1:** Effects of taurine on incidence of FLHS in laying hens.

Grouping	C	T1	T2	FLHS	FLHS + T1	FLHS + T2
Incidence of FLHS	4%	3%	3%	70%	10%	12%

**Table 2:** Ingredients of the diets and calculated nutrition levels for laying hens (%).

Ingredient, %	Standard Diet	High-Fat and Low-Protein Diet
Corn	61.5	61.5
Soybean meal	26.0	18.0
Soybean oil	0	8.0
Dicalcium Phosphate	1.2	1.2
Limestone	9.0	9.0
Premix	2.0	2.0
Salt	0.3	0.3
Total	100	100
Calculated composition		
Metabolizable energy, MJ/kg	11.09	12.85
Crude protein	16.85	13.51
Calcium	3.68	3.65
Available P	0.38	0.37
Lysine	0.93	0.69
Threonine (%)	0.67	0.52

**Table 3:** Primers for PCR analysis.

Gene	Primer Sequence
Gallus-Cpt1	Forward: 5′-GCCAAGTCGCTCGCTGATGAC-3′ Reverse: 5′-ACGCCTCGTAGGTCAGACAGAAC-3′
Gallus-Mfn1	Forward: 5′-TCCTGCTGCAACTCCAGAGA-3′ Reverse: 5′-ATCACTCCGCCAACAACGAT-3′
Gallus-Mfn2	Forward: 5′-CTGGCTCATGCCCTCCATCA-3′ Reverse: 5′-AATGCCAGGGCTGTCCATGA-3′
Gallus-Opa1	Forward: 5′-AGTCTGTGGAGCAGCAAGCA-3′ Reverse: 5′-TCCTGTCAAGCTCTCGCAAC-3′
Gallus-Fis1	Forward: 5′-TGTCTGTGGAGGACCTGCTGAAG-3′ Reverse: 5′-ACCAGGCACCAGGCGTACTC-3′
Gallus-Drp1	Forward: 5′- GAGCTCAACACTGCTATTTACG-3′ Reverse: 5′-TCAAAACAACCTTTCCGATCAC-3′
Gallus-Lc3I	Forward: 5′-TTACACCCATATCAGATTCTTG-3′ Reverse: 5′-ATTCCAACCTGTCCCTCA-3′
Gallus-Lc3II	Forward: 5′-CTTCTTCCTCCTGGTGAACG-3′ Reverse: 5′-GCACTCCGAAAGTCTCCTGA-3′
Gallus-P62	Forward: 5′-GACCCAGCCAAGACTACCAT-3′ Reverse: 5′-CAGAGGCATGTAGTTTCGGC-3′
Gallus-Beclin1	Forward: 5′-CGACTGGAGCAGGAAGAAG-3′ Reverse: 5′-TCTGAGCATAACGCATCTGG-3′
Gallus-Pgc1α	Forward: 5′-ATGTGTCGCCTTCTTGCTCTTC-3′ Reverse: 5′-GGACCTTGATCTTGACCTGGAA-3′
Gallus-Nrf1	Forward: 5′-AGCATTGAGGACTATCGTGAAG-3′ Reverse: 5′-TGGTTGTGGTCTGTTGCTGTGT-3′
Gallus-Nrf2	Forward: 5′-GCCTTCCTCTGCTGCCATTAGT-3′ Reverse: 5′-TGCCTTCAGTGTGCTTCTGGTT-3′
Gallus-Tfam	Forward: 5′-TTCCAGGAGGCTAAGGATGAG-3′ Reverse: 5′-CACTGCGACGGATGAGATCACT-3′
Gallus-β-actin	Forward: 5′-GAGAAATTGTGCGTGACATCA-3′ Reverse: 5′-CCTGAACCTCTCATTGCCA-3′
Homo-Sod2	Forward: 5′-GAGATGTTACACGCCCAGATAGC-3′ Reverse: 5′-AATCCCCAGCAGTGGAATAAGG-3′
Homo-Mfn1	Forward: 5′-TGCTGGCTAAGAAGGCGATTACTG-3′ Reverse: 5′-TGTCTCCGAGATAGCACCTCACC-3′
Homo-Mfn2	Forward: 5′-GTGCTTCTCCCTCAACTATGAC-3′ Reverse: 5′-ATCCGAGAGAGAAATGGAACTC-3
Homo-Opa1	Forward: 5′-TCTGCACACTCAGTTGAAGTAT-3′ Reverse: 5′-GCCTTTGTCATCTTTCTGCAAT-3′
Homo-Fis1	Forward: 5′-TGTCTGTGGAGGACCTGCTGAAG-3′ Reverse: 5′-ACCAGGCACCAGGCGTACTC-3′
Homo-Drp1	Forward: 5′-GAGCTCAACACTGCTATTTACG-3′ Reverse: 5′-TCAAAACAACCTTTCCGATCAC-3′
Homo-Pgc1α	Forward: 5′-CAGAGAGTATGAGAAGCGAGAG-3′ Reverse: 5′-AGCATCACAGGTATAACGGTAG-3
Homo-Nrf1	Forward: 5′-GCTACTTACACCGAGCATAGTA-3′ Reverse: 5′-CTCAAATACATGAGGCCGTTTC-3′
Homo-Nrf2	Forward: 5′-ACGGTATGCAACAGGACATTGAGC-3′ Reverse: 5′-TTGGCTTCTGGACTTGGAACCATG-3′
Homo-Tfam	Forward: 5′-AGCTCAGAACCCAGATGCAA-3′ Reverse: 5′-CCTGCCACTCCGCCCTATAA-3′
Homo-β-actin	Forward: 5′-GAGAGGGAAATCGTGCGTGACA-3′ Reverse: 5′-CGATAGTGACCTGACCGTCA-3′

## Data Availability

The data used to support the findings of this study are available upon request from the corresponding author.

## References

[1] Eslam M., Sanyal A.J., George J., Int Consensus P. (2020). MAFLD: A Consensus-Driven Proposed Nomenclature for Metabolic Associated Fatty Liver Disease. Gastroenterology.

[2] Marchesini G., Brizi M., Bianchi G., Tomassetti S., Bugianesi E., Lenzi M., McCullough A.J., Natale S., Forlani G., Melchionda N. (2001). Nonalcoholic fatty liver disease: A feature of the metabolic syndrome. Diabetes.

[3] Vuppalanchi R., Chalasani N. (2009). Nonalcoholic fatty liver disease and nonalcoholic steatohepatitis: Selected practical issues in their evaluation and management. Hepatology.

[4] Mancini M., Summers P., Faita F., Brunetto M.R., Callea F., De Nicola A., Di Lascio N., Farinati F., Gastaldelli A., Gridelli B. (2018). Digital liver biopsy: Bio-imaging of fatty liver for translational and clinical research. World J. Hepatol..

[5] Dai H., Chu L., Song S., Li W., Zhang L., Wu Z., Zeng J., Duan Q. (2009). Prevalence of and risk factors for fatty liver disease in a professional population of Wuhan, China. Public Health.

[6] Wong V.W., Chu W.C., Wong G.L., Chan R.S., Chim A.M., Ong A., Yeung D.K., Yiu K.K., Chu S.H., Woo J. (2012). Prevalence of non-alcoholic fatty liver disease and advanced fibrosis in Hong Kong Chinese: A population study using proton-magnetic resonance spectroscopy and transient elastography. Gut.

[7] Zhai H.L., Wang N.J., Han B., Li Q., Chen Y., Zhu C.F., Chen Y.C., Xia F.Z., Cang Z., Zhu C.X. (2016). Low vitamin D levels and non-alcoholic fatty liver disease, evidence for their independent association in men in East China: A cross-sectional study (Survey on Prevalence in East China for Metabolic Diseases and Risk Factors (SPECT-China)). Br. J. Nutr..

[8] Park J., Lee E.Y., Li J., Jun M.J., Yoon E., Ahn S.B., Liu C.L., Yang H.L., Rui F.J., Zou B.Y. (2021). NASH/Liver Fibrosis Prevalence and Incidence of Nonliver Comorbidities among People with NAFLD and Incidence of NAFLD by Metabolic Comorbidities: Lessons from South Korea. Dig. Dis..

[9] Friedman S.L., Neuschwander-Tetri B.A., Rinella M., Sanyal A.J. (2018). Mechanisms of NAFLD development and therapeutic strategies. Nat. Med..

[10] Meng J.C., Ma N., Liu H.L., Liu J., Liu J.X., Wang J.P., He X., Zhao X.H. (2021). Untargeted and targeted metabolomics profiling reveals the underlying pathogenesis and abnormal arachidonic acid metabolism in laying hens with fatty liver hemorrhagic syndrome. Poult. Sci..

[11] Chen W., Shi Y., Li G.Y., Huang C., Zhuang Y., Shu B., Cao X.H., Li Z.Q., Hu G.L., Liu P. (2021). Preparation of the peroxisome proliferator-activated receptor alpha polyclonal antibody: Its application in fatty liver hemorrhagic syndrome. Int. J. Biol. Macromol..

[12] Guo L.Y., Kuang J., Zhuang Y., Jiang J.L., Shi Y., Huang C., Zhou C.M., Xu P.Z., Liu P., Wu C. (2021). Serum Metabolomic Profiling to Reveal Potential Biomarkers for the Diagnosis of Fatty Liver Hemorrhagic Syndrome in Laying Hens. Front. Physiol..

[13] Rector R.S., Thyfault J.P., Uptergrove G.M., Morris E.M., Naples S.P., Borengasser S.J., Mikus C.R., Laye M.J., Laughlin M.H., Booth F.W. (2010). Mitochondrial dysfunction precedes insulin resistance and hepatic steatosis and contributes to the natural history of non-alcoholic fatty liver disease in an obese rodent model. J. Hepatol..

[14] Scorrano L. (2013). Keeping mitochondria in shape: A matter of life and death. Eur. J. Clin. Investig..

[15] Benard G., Bellance N., James D., Parrone P., Fernandez H., Letellier T., Rossignol R. (2007). Mitochondrial bioenergetics and structural network organization. J. Cell Sci..

[16] Delettre C., Lenaers G., Griffoin J.M., Gigarel N., Lorenzo C., Belenguer P., Pelloquin L., Grosgeorge J., Turc-Carel C., Perret E. (2000). Nuclear gene OPA1, encoding a mitochondrial dynamin-related protein, is mutated in dominant optic atrophy. Nat. Genet..

[17] Shirihai O.S., Song M., Dorn G.W. (2015). 2nd, How mitochondrial dynamism orchestrates mitophagy. Circ. Res..

[18] Dong H., Czaja M.J. (2011). Regulation of lipid droplets by autophagy. Trends Endocrinol. Metab..

[19] Jiang X., Tang X., Zhang P., Liu G., Guo H. (2014). Cyanidin-3-O-β-glucoside protects primary mouse hepatocytes against high glucose-induced apoptosis by modulating mitochondrial dysfunction and the PI3K/Akt pathway. Biochem. Pharmacol..

[20] Roysommuti S., Wyss J.M. (2014). Perinatal taurine exposure affects adult arterial pressure control. Amino Acids.

[21] Chang Y.Y., Chou C.H., Chiu C.H., Yang K.T., Lin Y.L., Weng W.L., Chen Y.C. (2011). Preventive effects of taurine on development of hepatic steatosis induced by a high-fat/cholesterol dietary habit. J. Agric. Food Chem..

[22] Murakami S., Fujita M., Nakamura M., Sakono M., Nishizono S., Sato M., Imaizumi K., Mori M., Fukuda N. (2016). Taurine ameliorates cholesterol metabolism by stimulating bile acid production in high-cholesterol-fed rats. Clin. Exp. Pharmacol. Physiol..

[23] Ito T., Yoshikawa N., Ito H., Schaffer S.W. (2015). Impact of taurine depletion on glucose control and insulin secretion in mice. J. Pharmacol. Sci..

[24] Chen X.C., Sebastian B.M., Tang H., McMullen M.M., Axhemi A., Jacobsen D.W., Nagy L.E. (2009). Taurine Supplementation Prevents Ethanol-Induced Decrease in Serum Adiponectin and Reduces Hepatic Steatosis in Rats. Hepatology.

[25] Ma Z., Zhang J., Ma H., Dai B., Zheng L., Miao J., Zhang Y. (2014). The influence of dietary taurine and reduced housing density on hepatic functions in laying hens. Poult. Sci..

[26] Tang R.Y., Yang Q.H., Lin S.M., Feng Y., Yang J.C., Lv Q.F., Wu G.F., Hu J.M., Hu J., Piao F., Schaffer S.W., ElIdrissi A., Wu J.Y. (2019). Preventive or Curative Administration of Taurine Regulates Lipid Metabolism in the Liver of Rats with Alcoholic Liver Disease. Taurine 11.

[27] Wu G.F., Tang R.Y., Yang J.C., Tao Y., Liu Z.Y., Feng Y., Lin S.M., Yang Q.H., Lv Q.F., Hu J.M., Marcinkiewicz J., Schaffer S.W. (2015). Taurine Accelerates Alcohol and Fat Metabolism of Rats with Alcoholic Fatty Liver Disease. Taurine 9.

[28] Wu G.F., Yang J.C., Lv H., Jing W.Y., Zhou J.Q., Feng Y., Lin S.M., Yang Q.H., Hu J.M. (2018). Taurine prevents ethanol-induced apoptosis mediated by mitochondrial or death receptor pathways in liver cells. Amino Acids.

[29] Gentile C.L., Nivala A.M., Gonzales J.C., Pfaffenbach K.T., Wang D., Wei Y., Jiang H., Orlicky D.J., Petersen D.R., Pagliassotti M.J. (2011). Experimental evidence for therapeutic potential of taurine in the treatment of nonalcoholic fatty liver disease. Am. J. Physiol. Regul. Integr. Comp. Physiol..

[30] Khalili Fard J., Hamzeiy H., Sattari M., Eghbal M.A. (2016). Protective Roles of N-acetyl Cysteine and/or Taurine against Sumatriptan-Induced Hepatotoxicity. Adv. Pharm. Bull..

[31] Jamshidzadeh A., Heidari R., Abasvali M., Zarei M., Ommati M.M., Abdoli N., Khodaei F., Yeganeh Y., Jafari F., Zarei A. (2017). Taurine treatment preserves brain and liver mitochondrial function in a rat model of fulminant hepatic failure and hyperammonemia. Biomed. Pharmacother..

[32] Heidari R., Babaei H., Eghbal M.A. (2014). Amodiaquine-induced toxicity in isolated rat hepatocytes and the cytoprotective effects of taurine and/or N-acetyl cysteine. Res. Pharm. Sci..

[33] Warskulat U., Borsch E., Reinehr R., Heller-Stilb B., Mönnighoff I., Buchczyk D., Donner M., Flögel U., Kappert G., Soboll S. (2006). Chronic liver disease is triggered by taurine transporter knockout in the mouse. FASEB J..

[34] Walzem R.L., Simon C., Morishita T., Lowenstine L., Hansen R.J. (1993). Fatty liver hemorrhagic syndrome in hens overfed a purified diet. Selected enzyme activities and liver histology in relation to liver hemorrhage and reproductive performance. Poult. Sci..

[35] Haghighi-Rad F., Polin D. (1981). The relationship of plasma estradiol and progesterone levels to the fatty liver hemorrhagic syndrome in laying hens. Poult. Sci..

[36] Stake P.E., Fredrickson T.N., Bourdeau C.A. (1981). Induction of fatty liver-hemorrhagic syndrome in laying hens by exogenous beta-estradiol. Avian. Dis..

[37] Rozenboim I., Mahato J., Cohen N.A., Tirosh O. (2016). Low protein and high-energy diet: A possible natural cause of fatty liver hemorrhagic syndrome in caged White Leghorn laying hens. Poult. Sci..

[38] Yang F., Ruan J., Wang T., Luo J., Cao H., Song Y., Huang J., Hu G. (2017). Improving effect of dietary soybean phospholipids supplement on hepatic and serum indexes relevant to fatty liver hemorrhagic syndrome in laying hens. Anim. Sci. J..

[39] Baylin A., Kabagambe E.K., Siles X., Campos H. (2002). Adipose tissue biomarkers of fatty acid intake. Am. J. Clin. Nutr..

[40] Yao H.R., Liu J., Plumeri D., Cao Y.B., He T., Lin L., Li Y., Jiang Y.Y., Li J., Shang J. (2011). Lipotoxicity in HepG2 cells triggered by free fatty acids. Am. J. Transl. Res..

[41] Malhi H., Bronk S.F., Werneburg N.W., Gores G.J. (2006). Free fatty acids induce JNK-dependent hepatocyte lipoapoptosis. J. Biol. Chem..

[42] Huang Z.J., Wang M.Y., Liu L., Peng J.F., Guo C.X., Chen X.P., Huang L., Tan J.Q., Yang G.P. (2019). Transcriptional Repression of CYP3A4 by Increased miR-200a-3p and miR-150-5p Promotes Steatosis in vitro. Front. Genet..

[43] Tan J.R., Xu J.H., Wei G.H., Zhang L.J., Sun L.E., Wang G.Y., Li F., Jiang F.X. (2019). HNF1 alpha Controls Liver Lipid Metabolism and Insulin Resistance via Negatively Regulating the SOCS-3-STAT3 Signaling Pathway. J. Diabetes Res..

[44] Zhang S.R., Mao Y.Q., Fan X.M. (2018). Inhibition of ghrelin o-acyltransferase attenuated lipotoxicity by inducing autophagy via AMPK-mTOR pathway. Drug Des. Dev. Ther..

[45] Chu J.H., Wang H., Ye Y., Chan P.K., Pan S.Y., Fong W.F., Yu Z.L. (2011). Inhibitory effect of schisandrin B on free fatty acid-induced steatosis in L-02 cells. World J. Gastroenterol..

[46] Kalaz E.B., Çoban J., Aydın A.F., Doğan-Ekici I., Doğru-Abbasoğlu S., Öztezcan S., Uysal M. (2014). Carnosine and taurine treatments decreased oxidative stress and tissue damage induced by D-galactose in rat liver. J. Physiol. Biochem..

[47] Park T., Lee K. (1998). Dietary taurine supplementation reduces plasma and liver cholesterol and triglyceride levels in rats fed a high-cholesterol or a cholesterol-free diet. Adv. Exp. Med. Biol..

[48] Choi M.J., Kim J.H., Chang K.J. (2006). The effect of dietary taurine supplementation on plasma and liver lipid concentrations and free amino acid concentrations in rats fed a high-cholesterol diet. Adv. Exp. Med. Biol..

[49] Murakami S., Ono A., Kawasaki A., Takenaga T., Ito T. (2018). Taurine attenuates the development of hepatic steatosis through the inhibition of oxidative stress in a model of nonalcoholic fatty liver disease in vivo and in vitro. Amino Acids.

[50] Wu Q., Tang H.Q., Wang H.B. (2019). The anti-oxidation and mechanism of essential oil of paederia scandens in the NAFLD model of chicken. Animals.

[51] Jiang Y.Z., Chen L., Wang H., Narisi B., Chen B. (2015). Li-Gan-Shi-Liu-Ba-Wei-San improves non-alcoholic fatty liver disease through enhancing lipid oxidation and alleviating oxidation stress. J. Ethnopharmacol..

[52] Doc Z., Kapusta E., Formicki G., Martiniakova M., Omelka R. (2019). Effect of Taurine on Ethanol-Induced Oxidative Stress in Mouse Liver and Kidney. Chin. J. Physiol..

[53] Zhu W.H., Chen S.W., Chen R.G., Peng Z.Q., Wan J., Wu B.Y. (2017). Taurine and tea polyphenols combination ameliorate nonalcoholic steatohepatitis in rats. BMC Complement. Altern. Med..

[54] Rines A.K., Ardehali H. (2013). Transition metals and mitochondrial metabolism in the heart. J. Mol. Cell. Cardiol..

[55] Sanyal A.J., Campbell-Sargent C., Mirshahi F., Rizzo W.B., Contos M.J., Sterling R.K., Luketic V.A., Shiffman M.L., Clore J.N. (2001). Nonalcoholic steatohepatitis: Association of insulin resistance and mitochondrial abnormalities. Gastroenterology.

[56] Wei Y., Rector R.S., Thyfault J.P., Ibdah J.A. (2008). Nonalcoholic fatty liver disease and mitochondrial dysfunction. World J. Gastroenterol..

[57] Pessayre D., Fromenty B. (2005). NASH: A mitochondrial disease. J. Hepatol..

[58] Du J., Zhang X., Han J., Man K., Zhang Y., Chu E.S., Nan Y., Yu J. (2017). Pro-Inflammatory CXCR3 Impairs Mitochondrial Function in Experimental Non-Alcoholic Steatohepatitis. Theranostics.

[59] Rolo A.P., Teodoro J.S., Palmeira C.M. (2012). Role of oxidative stress in the pathogenesis of nonalcoholic steatohepatitis. Free. Radic. Biol. Med..

[60] Lim C.Y., Jun D.W., Jang S.S., Cho W.K., Chae J.D., Jun J.H. (2010). Effects of carnitine on peripheral blood mitochondrial DNA copy number and liver function in non-alcoholic fatty liver disease. Korean J. Gastroenterol..

[61] Serviddio G., Bellanti F., Tamborra R., Rollo T., Capitanio N., Romano A.D., Sastre J., Vendemiale G., Altomare E. (2008). Uncoupling protein-2 (UCP2) induces mitochondrial proton leak and increases susceptibility of non-alcoholic steatohepatitis (NASH) liver to ischaemia-reperfusion injury. Gut.

[62] Sihali-Beloui O., El-Aoufi S., Maouche B., Marco S. (2016). Psammomys obesus, a unique model of metabolic syndrome, inflammation and autophagy in the pathologic development of hepatic steatosis. C. R. Biol..

[63] Rautou P.E., Mansouri A., Lebrec D., Durand F., Valla D., Moreau R. (2010). Autophagy in liver diseases. J. Hepatol..

[64] Amir M., Czaja M.J. (2011). Autophagy in nonalcoholic steatohepatitis. Expert Rev. Gastroenterol. Hepatol..

[65] Legros F., Lombes A., Frachon P., Rojo M. (2002). Mitochondrial fusion in human cells is efficient, requires the inner membrane potential, and is mediated by mitofusins. Mol. Biol. Cell.

[66] Olichon A., Baricault L., Gas N., Guillou E., Valette A., Belenguer P., Lenaers G. (2003). Loss of OPA1 perturbates the mitochondrial inner membrane structure and integrity, leading to cytochrome c release and apoptosis. J. Biol. Chem..

[67] Fekkes P., Shepard K.A., Yaffe M.P. (2000). Gag3p, an outer membrane protein required for fission of mitochondrial tubules. J. Cell Biol..

[68] Tong W., Ju L., Qiu M., Xie Q., Chen Y., Shen W., Sun W., Wang W., Tian J. (2016). Liraglutide ameliorates non-alcoholic fatty liver disease by enhancing mitochondrial architecture and promoting autophagy through the SIRT1/SIRT3-FOXO3a pathway. Hepatol. Res..

[69] Gong F., Gao L., Ding T. (2019). IDH2 protects against nonalcoholic steatohepatitis by alleviating dyslipidemia regulated by oxidative stress. Biochem. Biophys. Res. Commun..

[70] Sasi U.S.S., Sindhu G., Raghu K.G. (2020). Fructose-palmitate based high calorie induce steatosis in HepG2 cells via mitochondrial dysfunction: An in vitro approach. Toxicol. Vitr..

[71] Eid N., Ito Y., Otsuki Y. (2016). Triggering of Parkin Mitochondrial Translocation in Mitophagy: Implications for Liver Diseases. Front. Pharmacol..

[72] Liu P.Y., Lin H.K., Xu Y.Y., Zhou F., Wang J., Liu J.J., Zhu X.H., Guo X.P., Tang Y.H., Yao P. (2018). Frataxin-Mediated PINK1-Parkin-Dependent Mitophagy in Hepatic Steatosis: The Protective Effects of Quercetin. Mol. Nutr. Food Res..

[73] Gonçalves I.O., Passos E., Diogo C.V., Rocha-Rodrigues S., Santos-Alves E., Oliveira P.J., Ascensão A., Magalhães J. (2016). Exercise mitigates mitochondrial permeability transition pore and quality control mechanisms alterations in nonalcoholic steatohepatitis. Appl. Physiol. Nutr. Metab..

[74] Martinez-Vicente M., Talloczy Z., Wong E., Tang G., Koga H., Kaushik S., de Vries R., Arias E., Harris S., Sulzer D. (2010). Cargo recognition failure is responsible for inefficient autophagy in Huntington’s disease. Nat. Neurosci..

[75] Kim K.H., Lee M.S. (2014). Autophagy as a crosstalk mediator of metabolic organs in regulation of energy metabolism. Rev. Endocr. Metab. Disord..

[76] Begriche K., Sutton G.M., Fang J., Butler A.A. (2009). The role of melanocortin neuronal pathways in circadian biology: A new homeostatic output involving melanocortin-3 receptors?. Obes. Rev..

[77] Schmittgen T.D., Livak K.J. (2008). Analyzing real-time PCR data by the comparative CT method. Nat. Protoc..

